# Low-Volume and High-Intensity Aerobic Interval Training May Attenuate Dysfunctional Ventricular Remodeling after Myocardial Infarction: Data from the INTERFARCT Study

**DOI:** 10.31083/j.rcm2401020

**Published:** 2023-01-11

**Authors:** Gualberto Rodrigo Aispuru-Lanche, Monica Gallego-Muñoz, Jon Ander Jayo-Montoya, Beatriz Villar-Zabala, Sara Maldonado-Martín

**Affiliations:** ^1^Department of Physiology, Faculty of Medicine, University of the Basque Country (UPV/EHU), Leioa, 48940 Bizkaia, Basque Country, Spain; ^2^Primary Care Administration of Burgos, Salud Castilla y Leon (Sacyl), 09267 Burgos, Spain; ^3^Department of Physiology, Faculty of Pharmacy, University of the Basque Country (UPV/EHU), Vitoria-Gasteiz, 01006 Alava/Araba, Basque Country, Spain; ^4^Department of Physical Education and Sport, Faculty of Education and Sport, Physical Activity and Sport Sciences Section, University of the Basque Country (UPV/EHU), 01007 Vitoria-Gasteiz, Alava, Basque Country, Spain; ^5^GIzartea, Kirola eta Ariketa Fisikoa Ikerkuntza Taldea (GIKAFIT), Society, Sports, and Physical Exercise Research Group, Department of Physical Education and Sport, Faculty of Education and Sport-Physical Activity and Sport Sciences Section, University of the Basque Country (UPV/EHU), Vitoria-Gasteiz, 01007 Araba/Álava, Basque Country, Spain; ^6^Bioaraba Health Research Institute, Physical Activity, Exercise, and Health group, 01007 Vitoria-Gasteiz, Basque Country, Spain

**Keywords:** high-intensity interval training, cardiac remodeling, coronary heart disease, secondary prevention

## Abstract

**Background:**

Aerobic high-intensity interval training (HIIT) has demonstrated benefits for ventricular remodeling after myocardial infarction (MI) through various mechanisms. Despite this, the optimal training volume is not well known. The present study aimed to assess the effects of different (low vs. high volume) aerobic HIIT compared to an attentional control (AC) group on echocardiographic and biochemical indicators of left ventricular (LV) remodeling in adults after MI.

**Methods:**

Randomized clinical trial conducted on post-MI patients with preserved ventricular function. Participants were assigned to three study groups. Two groups performed HIIT 2 d/week, one group with low-volume HIIT (20 min, n = 28) and another with high-volume HIIT (40 min, n = 28). A third group was assigned to AC (n = 24) with recommendations for unsupervised aerobic training. Left ventricular echocardiographic parameters and cardiac biomarker levels (N-terminal pro-b-type natriuretic peptide, NT-proBNP; soluble growth stimulation expressed gene 2, ST2; troponin T; and creatine kinase) were assessed at baseline and after the intervention (16 weeks).

**Results:**

Eighty participants (58.4 ± 8.3 yrs, 82.5% male) were included. Both low- and high-volume HIIT showed increases (*p *< 0.05) in left ventricular end-diastolic diameter (1.2%, 2.6%), and volume (1.1%, 1.3%), respectively. Interventricular septal and posterior walls maintained their thickness (*p* = 0.36) concerning the AC. Significant (*p *< 0.05) gain in diastolic function was shown with the improvements in E (–2.1%, –3.3%), e’ waves (2.2%, 5.5%), and the deceleration time (2.1%, 2.9%), and in systolic function with a reduction in global longitudinal strain (–3.2%, –4.7%), respectively. Significant (*p *< 0.05) reductions of N-terminal pro-B-type natriuretic peptide (NT-proBNP) (–4.8%, –11.1%) and of ST2 (–21.7%, –16.7%)were found in both HIIT groups respectively compared to the AC group. Creatine kinase elevation was shown only in high-volume HIIT (19.3%, *p *< 0.01).

**Conclusions:**

Low-volume HIIT is proposed as a clinically time-efficient and safer strategy to attenuate dysfunctional remodeling by preventing wall thinning and improving LV function in post-MI patients.

## 1. Introduction

Myocardial infarction (MI) is a relevant cause of morbidity and mortality in the Western world [1]. The lack of blood flow to a part of the heart causes an injury to the myocardium that predisposes to thinning of the wall and dilatation of the ventricular cavities [2]. Consequently, remodeling is the adaptive or maladaptive response to cardiac overload resulting in echocardiographic changes in the size and function of the heart [3]. Studies of adverse ventricular remodeling post-MI have gained clinical interest to try to prevent these outcomes [4, 5]. Concerning this, cardiac biomarkers have emerged as a link to understanding complex cardiac adaptation [6]. Traditional markers are N-terminal pro-B-type natriuretic peptide (NT-proBNP), an indicator of cardiomyocyte stretching and the progression to chronic heart failure; troponin T, a marker of myocardial injury [7]; and creatine kinase, a marker of musculoskeletal damage. The growth stimulation expressed gene 2 (ST2), a member of interleukine-1 family receptors, is expressed on cardiomyocytes as a response to an increase in biomechanical stress and could determine early negative cardiac remodeling [8]. In general, an excessive increase in these biomarker levels is accompanied by a deleterious effect that could predict the development of negative left ventricular (LV) remodeling and heart failure in the post-MI period [9].

Exercise-based cardiac rehabilitation has proven to be an important tool to reduce high cardiovascular risk in patients after MI [10]. The benefits of aerobic exercise have been studied and shown to improve both cardiovascular and non-cardiovascular parameters [11]. Growing evidence is demonstrating superior patient outcomes resulting from aerobic high-intensity interval training (HIIT, *i.e.*, repeated bouts of high-intensity effort followed by varied recovery times) compared to aerobic moderate-intensity continuous training in patients with coronary artery disease (CAD) [12]. This superiority of the HIIT programs in post-MI patients has mainly been related to cardiorespiratory fitness [13] and cardiometabolic health [14]. Thus, previous analyses from the INTERFARCT study have shown that low-volume HIIT (*i.e.*, less than 10 min at high-intensity effort in the same exercise session) is as effective and time-efficient as a training strategy to achieve improvements in cardiorespiratory fitness, and body composition, and chronotropic responses as high-volume HIIT [15, 16]. However, while HIIT has shown reversible LV remodeling in patients with post-MI heart failure (up to 20% of cases) [17, 18], in the absence of cardiac failure the effect on LV structure and function is not yet clear [19, 20]. Therefore, given the considerable number of patients with preserved ventricular function after MI, the need to prevent progression to heart failure [21], and the increased use of HIIT in cardiac rehabilitation programs for patients with CAD, it is necessary to know the appropriate volume of this type of training and its effects on different parameters associated with ventricular remodeling.

This research project aimed to assess the effects of different (low- *vs*. high-volume) aerobic HIIT compared to an attentional control (AC) group on echocardiographic and biochemical indicators of LV remodeling in adults after MI. We hypothesized that HIIT and specifically low-volume could be a time-effective strategy to prevent dysfunctional ventricular remodeling and could provide clues for the adequate use of this training in patients with CAD without heart failure.

## 2. Material and Methods

### 2.1 Study Design

**Fig. 1.**
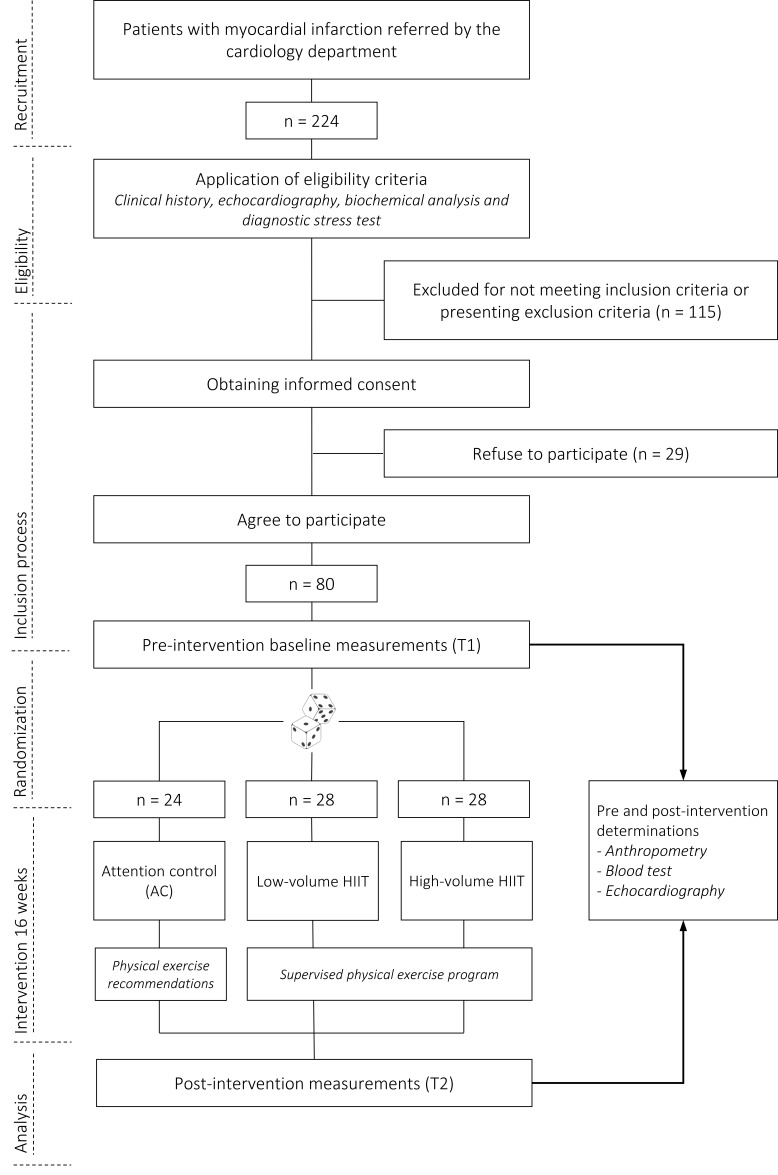
**Flow chart of the INTERFARCT study from enrollment to end of intervention with measurements used to assess the impact on the ventricular remodeling of two volumes (low *vs.* high) of HIIT compared to a control group in individuals with myocardial infarction**. Abbreviations: AC, attention control group; HIIT, high-intensity interval training.

A detailed description of study design, eligibility, and participants of the study on different aerobic INTERval exercise training volumes, high *vs.* low, in people after a myocardial inFARCTion (INTERFARCT, ClinicalTrials.gov: NCT02876952) has been previously published [22]. Briefly, patients after MI with preserved systolic function referred for cardiac rehabilitation were randomly divided into three groups: assigned either to the AC group or one of the two supervised HIIT groups two days/week for 16 weeks: low-volume HIIT (20 min) and high-volume HIIT (40 min). A schematic presentation of the study flow of participants is outlined in Fig. 1 and the inclusion and exclusion criteria for the INTERFARCT study are shown in Table 1.

**Table 1.** **Inclusion and exclusion criteria for the INTERFARCT study**.

Inclusion criteria	
	- Spontaneous MI, with and without ST elevation
	- Effective revascularization treatment (coronary artery bypass grafting or percutaneous coronary intervention)
	- Age ≥18 years, clinically stable, and sinus rhythm
	- Between one and six months after MI
	- Left ventricular ejection fraction >50%
	- Time availability (45 min, two days a week for 16 weeks) to carry out the exercise program
Exclusion criteria	
	- Unstable coronary artery disease, uncontrolled hypertension, malignant ventricular arrhythmia, atrial fibrillation, exercise-induced ischemia, and ventricular failure during exercise
	- Other significant medical conditions including, but not limited to: chronic or recurrent respiratory, gastrointestinal, neuromuscular, neurological, or psychiatric conditions; musculoskeletal problems interfering with exercise; severe kidney disease (creatinine clearance <30 mL/min); anemia (hemoglobin <12 g/dL); bleeding disorders; systemic malignancies in the past five years; type 1 diabetes; moderate to severe peripheral artery disease (> IIa in Fontaine’s classification); any other medical condition or disease that is life-threatening or that can interfere with or be aggravated by exercise
	- Any other co-morbidity with life expectancy <1 year
	- Could not perform a valid baseline exercise test
	- Obesity >35 kg/$m^{2}$
	- Pregnancy or breastfeeding
	- Plans to be out of the city for >2 weeks

### 2.2 Measurements

Anthropometric measurements, echocardiography, and blood analyses were taken before (T1) and after (T2) the 16-week intervention period. The tests were performed with a minimum of eight hours of fasting and 12 hours of abstinence from alcohol, caffeine, and vitamins. Post-intervention determinations were made the following week after the end of the intervention period.

Transthoracic 2D echocardiography was performed by two cardiology specialists blinded to the patient’s group assignment. These occurred at different times to assess the reproducibility of different measurements by test-retest [23]. The inter-observer average measurements were used in the statistical analysis and the reproducibility was expressed by the mean percent error (mean error). Mean error was derived as the absolute difference between the two observations, divided by the mean of the observations, and presented as a percentage [24]. Values <10% were considered to have adequate robustness to be included in the analysis. Participants were examined at rest using a Vivid 9 cardiac ultrasound system with a 7.5-MHz transducer (GE Healthcare) according to standard procedures for evaluating left ventricle dimensions and systolic and diastolic functions [25]. Conventional B-mode, color Doppler, pulsed, and continuous-wave Doppler images were acquired in still or cine format using electrocardiogram-gating. All data was obtained as the average of 3–5 heartbeats [26, 27]. Adequate acquisition of echocardiographic images was very important for this study and was considered an eligibility criterion. Participants with inadequate availability to measure LV characteristics were not included. More detail on the ultrasonographic determinations has been previously published in the protocol of the INTERFARCT study [22].

A blood sample was obtained at each stage of the protocol in ethylenediamine tetraacetic acid- anticoagulated plastic tubes and serum were isolated within one hour of collection. The blood sample in T2 was obtained 48 hours after finishing the last HIIT training. Aliquots of plasma were then stored at –80 °C until thawed for the determination of biomarkers. Soluble ST2 was measured using a quantitative sandwich monoclonal enzyme-linked immunosorbent assay (Presage sST2 Assay; Critical Diagnostics) [28]. NT- proBNP and high-sensitivity troponin T (hs-TnT) were measured through the electrochemiluminescence monoclonal method (proBNP assay and Elecsys hs-TnT, Roche Diagnostics respectability). Creatine kinase as an indicator of nonspecific muscle damage was measured by an AU 2700 device (Beckman Coulter, Inc., Fullerton, CA, USA).

### 2.3 Intervention

Participants (n = 80) were randomly assigned to one of three intervention groups; two groups performed a supervised HIIT training program and one group followed unsupervised exercise recommendations within the AC. The intensity of the exercise for each participant in the three groups was individually scheduled based on cardiorespiratory fitness and ventilatory thresholds (VT1 and VT2), which were determined from the peak oxygen consumption (${\mathrm{VO}}_{\text{2peak}}$) obtained by an ergospirometry (Lode Excalibur SportCycle, Partnumber: 925909, 2007, Groningen, The Netherlands) and expired gas analysis (Ergocard Medisoft SS, Belgium Ref. USM001 V1.0). Based on the identification of the two VT, the three intensity ranges of exercise were determined: (R1) light to moderate intensity with heart rate (HR) values and below VT1; (R2) moderate to high intensity with HR values between VT1 and VT2; and (R3) high to severe intensity with HR values from VT2 to the maximum HR achieved in the cardiopulmonary stress test. More specific information regarding the exercise cardiopulmonary test and ventilatory threshold assessment have been previously published [22].

The supervised HIIT groups exercised two days per week in one of two randomly assigned interval training programs (alternating R2 with R3): (1) low-volume HIIT group, with less than 10 min at R3 each session, with a total volume of 20 min; (2) high-volume HIIT group with more than 10 min at R3 each session and gradually increased from 20 to 40 min for the total volume. Supervised groups performed exercise sessions on two non-consecutive days of the week (one day on the treadmill, and the second day on the cycle ergometer) for 16 weeks (32 sessions). Training intensity was controlled by monitoring beat-to-beat HR (Polar Electro, Kempele, Finland) and through the rate of perceived exertion using the original Börg scale (6–20 points). The justification for a mixed training model alternating a treadmill and a cycle ergometer was to avoid the great osteoarticular impact that two days on a treadmill would generate, considering the high intensity of the program and the high prevalence of overweight among the participants. To achieve the “target” HR goals in each range (R2 and R3), the intensity was individually adapted through speed and incline on the treadmill and through watts on the cycle ergometer. The specific protocols of the HIIT programs have been previously published [22]. Briefly, on the treadmill, there were intervals of 4 min at R3 followed by 3 min at R2, and on the exercise bike intervals of 30 s at R3 followed by 60 s at R2.

The AC received individualized recommendations to perform physical exercise without supervision for 16 weeks. They were advised to engage in at least 30 min of moderate-intensity aerobic physical activity (*i.e.*, walking, jogging, cycling, swimming) 5 to 7 days per week [29]. Each participant received training information with their HR intensity domains calculated by ergospirometry for self-control. All participants had individual nutritional counseling every two weeks to help them increase their adherence to a cardio-healthy diet (Mediterranean Diet) [30]. This was performed by a nurse specialized in Family and Community Medicine with specific training in nutrition for high cardiovascular-risk populations, who was blinded to the randomized intervention groups.

### 2.4 Statistical Analysis

Statistical analysis of the results was performed using the statistical package IBM SPSS Statistics 22.0 (Armonk, NY, USA: IBM Corp.). For comparisons between groups at baseline and pre-post means, a one-way analysis of variance (ANOVA) or nonparametric Kruskal-Wallis method and Chi-square test were used. Furthermore, to evaluate the effects of the interventions on the outcomes of the primary (left ventricle diastolic diameter) and secondary variables, a linear regression model with ANOVA was used. Pre-post differences (delta, Δ) with their relative values (%Δ) of each variable for each group (AC, low-volume HIIT, and high-volume HIIT) were calculated. The Shapiro-Wilk test was used to evaluate the normal distribution of the variables, fulfilling the assumption of normality. Bonferroni correction was used, and the significance level was calculated: time (analysis of the significance of the main effect within the participants of the same group), group (analysis of the significance of the main effect among groups), and analysis of the significance that determines the interaction of the two main effects found (group*time). A Pearson bivariate correlation analysis was performed to detect the correlation between continuous variables with physiological coherence. Data were analyzed according to the intention-to-treat principle. Data are expressed as mean ± standard deviation (SD) for continuous variables, while for categorical variables it is presented with frequencies and percentages. The level of significance was established at 5% (α = 0.05).

## 3. Results

### 3.1 Study Population

A total of 224 post-MI patients were consecutively referred for two years to eligibility assessment (March 2016 to February 2018). Of these enrolled patients, 115 were excluded and the other 29 refused to participate (Fig. 1). There were five patients among those excluded who presented poor echocardiographic image acquisition in addition to other criteria. Finally, 80 participants were randomly assigned to one of the three groups (AC: n = 24, low-volume HIIT: n = 28, high-volume HIIT: n = 28). The baseline demographic and clinical characteristics of the study population are summarized in Table 2. The male:female ratio was 4.7:1 and there were no significant differences among groups regarding demographic data, event details, medical conditions, and medication (*p *> 0.05).

**Table 2.** **Baseline demographic and clinical characteristics of the participants**.

		Total group		AC		HIIT groups				*p* value
						Low-volume		High-volume		
Sample size, n		80		24		28		28		
Male gender		66	(82.5)	19	(79.2)	24	(85.7)	23	(82.1)	0.82
Age, yr		58.4	±8.3	57.0	±7.2	59.0	±9.6	58.9	±8.0	0.63
Body mass, kg		83.4	±17.8	79.2	±17.0	85.2	±20.5	85.4	±16.5	0.39
BMI, kg/$m^{2}$		29.9	±6.6	28.1	±5.4	30.5	±6.8	30.7	±4.9	0.21
Rest SBP, mmHg		128.8	±12.0	126.0	±12.1	127.0	±13.4	133.3	±10.4	0.06
Rest DBP, mmHg		78.1	±8.2	77.4	±8.1	77.6	±7.3	79.2	±9.1	0.68
Rest HR, beats/min		70.3	±8.6	69.6	±8.7	65.8	±12.2	65.7	±9.2	0.31
Peak HR, beats/min		136.2	±22.9	141.9	±25.9	134.1	±20.8	132.7	±22.3	0.47
${\mathrm{VO}}_{\text{2peak}}$, mL•${\mathrm{kg}}^{-1}$•$\min^{-1}$		23.5	±6.6	27.6	±8.6	23.1	±8.1	23.2	±5.2	0.24
Peak RER		1.2	±0.1	1.2	±0.1	1.2	±0.1	1.2	±0.2	0.47
MET		7.0	±2.1	7.8	±2.4	6.6	±2.3	6.7	±1.4	0.24
Event characteristics										
STEMI		60	(75.0)	19	(79.2)	21	(75.0)	20	(71.4)	0.93
Infarct-related vessels										
	Anterior descending	40	(50.0)	13	(54.2)	12	(42.9)	15	(43.6)	0.77
	Circumflex	17	(21.3)	5	(20.8)	7	(25.0)	5	(17.9)	0.83
	Right coronary	23	(28.8)	6	(25.0)	9	(32.1)	8	(28.6)	0.67
Primary PCI		78	(97.5)	23	(95.8)	28	(100)	27	(96.4)	0.96
Time post-MI (days)		35.7	±7.2	35.8	±8.1	35.4	±7.9	36.1	±6.5	0.95
Cardiovascular risk factor, %										
Hypertension		64	(80.0)	19	(79.2)	23	(82.1)	22	(78.6)	0.23
Dyslipidemia		70	(87.5)	21	(87.5)	24	(85.7)	25	(89.3)	0.92
Diabetes mellitus		23	(28.8)	6	(25.0)	8	(28.6)	9	(32.1)	0.85
Smoking										
	Ex-smoker	63	(78.8)	18	(75.0)	22	(78.6)	23	(82.1)	0.82
	Smoker	7	(8.7)	2	(8.3)	3	(10.7)	2	(7.1)	0.80
Family predisposition		10	(12.5)	3	(12.5)	4	(14.3)	3	(10.7)	0.92
Sleep apnea syndrome		9	(11.2)	2	(8.3)	3	(10.7)	4	(14.2)	0.91
Medication, %										
Aspirin		75	(93.8)	22	(91.7)	27	(96.4)	26	(92.9)	0.77
Antithrombotics		44	(55.0)	13	(54.2)	16	(57.1)	15	(53.6)	0.96
Oral anticoagulants		3	(3.8)	1	(4.2)	1	(3.6)	1	(3.6)	0.99
RAAS inhibitors		70	(87.5)	20	(83.3)	24	(85.7)	26	(92.9)	0.54
Beta-blockers		72	(90.0)	21	(87.5)	25	(89.3)	26	(92.9)	0.80
CCB		16	(20.0)	5	(20.8)	6	(21.4)	5	(17.9)	0.94
Diuretics		18	(22.5)	5	(20.8)	6	(21.4)	7	(25.0)	0.92
Lipid-lowering therapy		78	(97.5)	23	(95.8)	28	(100)	27	(96.4)	0.57
	Statins	76	(95.0)	22	(91.7)	28	(100)	26	(92.9)	0.59
Antidiabetic medication		19	(23.8)	5	(20.8)	6	(21.4)	8	(28.6)	0.76
	SGLT2 inhibitor	9	(11.2)	2	(8.3)	3	(10.7)	4	(14.3)	0.71

Data are expressed as mean ± SD, dichotomous variables are expressed as numbers and percentages (%). Abbreviations: AC, attention control group; BMI, body mass index; CCB, calcium channel blocker; DBP, diastolic blood pressure; HIIT, high-intensity interval training; HR, heart rate; MET: metabolic equivalent of task; PCI, percutaneous coronary intervention; RAAS inhibitors, inhibitors of the renin-angiotensin-aldosterone system; RER, respiratory exchange ratio; SBP, systolic blood pressure; SGLT2 inhibitor, sodium-glucose cotransporter-2 inhibitors STEMI, ST-elevation myocardial infarction; ${\mathrm{VO}}_{\text{2peak}}$, peak oxygen uptake. Statistics: One-way analysis of variance (ANOVA) or nonparametric Kruskal-Wallis method and Chi-square test were used for comparisons between groups at baseline (*p*).

Relevant adverse events were not reported during the intervention and the mean exercise adherence reached 93.7% of the 32 scheduled sessions. The mean adherence to the target intensity of all the patients during the intervention sessions was 89.9 ± 7.2% and 88.4 ± 8.3% in the low- and high-volume HIIT, respectively (*p *= 0.889). Inhibitors of the renin-angiotensin-aldosterone system doses were changed in five participants across the study: reduced in three patients in the low-volume HIIT group and two patients in the high-volume HIIT group. Beta-blocker doses were not changed in any of the participants. There was no dropout of participants during the different periods of the study, ending with the same initial sample recruited (n = 80).

### 3.2 Left Ventricular Echocardiography 

Echocardiographic parameters of LV geometry, systolic and diastolic function in the three groups at T1 and T2 are summarized in Table 3. No baseline difference was detected among groups regarding echocardiographic values and the measurements had acceptable inter-operator error bias. A significant reduction of –7.2% (*p* = 0.004) in the interventricular septum thickness and of –6.3% (*p* = 0.04) in the posterior wall thickness was observed in the AC group at the end of the study (T2). In contrast, these ventricular walls in the two HIIT groups remained unchanged but prone to thickening. Left ventricle end-diastolic diameter (LVEDD) and volume (LVEDV) increased significantly in all groups with a slight trend toward a greater increase in high-volume HIIT (Table 3).

**Table 3.** **Left ventricular geometry, systolic and diastolic function adaptations to different volumes of aerobic high-intensity interval training in post-myocardial infarction patients**.

		AC			HIIT groups				*p *values		
					Low-volume		High-volume		Time	Group	Interaction Group*Time
Sample size, n		24			28		28				
LV geometry											
ISWT, mm	T1	9.7		±1.3 (1.6)	9.5	±1.5 (1.9)	9.7	±1.6 (1.0)			
	T2	9.0		±2.0^**^ (2.1)	9.8	±1.6 (1.7)	9.9	±1.0 (2.2)	0.03	<0.01	<0.01
PWT, mm	T1	9.5		±1.2 (3.5)	9.4	±1.6 (2.7)	9.5	±1.5 (3.3)			
	T2	8.9		±1.4^**^ (3.1)	9.5	±1.5 (2.9)	9.6	±1.4 (2.5)	0.05	0.04	0.03
LVMI, g/$m^{2}$	T1	68.8		±14.6 (4.0)	69.5	±13.4 (3.2)	68.6	±12.2 (3.7)			
	T2	68.0		±13.7 (5.6)	71.0	±14.5^**^ (4.1)	72.5	±12.6^**^ (3.5)	0.15	0.16	0.06
LVEDD, mm	T1	52.3		±5.0 (6.3)	51.8	±4.4 (7.1)	52.7	±6.4 (5.3)			
	T2	53.5		±${\mathrm{5.4}}^{*}$ (7.2)	52.6	±${\mathrm{4.6}}^{*}$ (6.5)	54.2	±7.1^**^ (7.5)	0.03	0.04	0.85
LVEDV, mL	T1	151.3		±19.5 (2.5)	150.9	±19.5 (1.2)	152.1	±19.6 (1.8)			
	T2	152.7		±${\mathrm{19.6}}^{*}$ (4.7)	152.5	±${\mathrm{20.6}}^{*}$ (3.3)	154.1	±${\mathrm{23.0}}^{*}$ (3.1)	0.04	0.03	0.25
LVESV, mL	T1	57.3		±9.5 (8.1)	58.4	±9.7 (8.2)	57.9	±9.7 (7.7)			
	T2	58.9		±9.7 (4.3)	59.6	±${\mathrm{10.2}}^{*}$ (7.0)	61.0	±10.8^**^ (6.4)	0.67	0.08	0.61
Systolic function											
LVEF, %	T1	59.1		±6.0 (3.1)	59.4	±6.8 (2.9)	60.2	±6.2 (3.4)			
	T2	58.1		±6.1 (4.5)	61.7	±${\mathrm{6.8}}^{*}$ (3.8)	62.8	±${\mathrm{6.8}}^{*}$ (2.7)	0.71	0.68	0.79
SVI, mL/$m^{2}$	T1	60.4		±10.4 (5.2)	62.8	±10.3 (5.0)	62.1	±10.4 (6.1)			
	T2	60.0		±7.6 (5.5)	63.2	±6.9 (5.5)	64.6	±6.6 (4.2)	0.66	0.45	0.74
CO, L·$\min^{-1}$	T1	4.6		±0.5 (4.7)	4.6	±0.5 (4.4)	4.6	±0.5 (3.9)			
	T2	4.6		±0.4 (4.5)	4.7	±0.5 (4.2)	4.7	±0.6 (4.2)	0.51	0.77	0.86
GLS, %	T1	–18.4		±2.9 (9.2)	–18.7	±3.2 (8.3)	–19.1	±3.4 (9.9)			
	T2	–18.1		±3.3 (8.7)	–19.3	±2.7^**^ (9.1)	–20.0	±3.5^**^ (8.0)	0.03	0.02	0.04
S’, cm/s	T1	6.0		±1.7 (4.3)	6.2	±1.9 (2.2)	6.1	±1.9 (4.2)			
	T2	6.1		±1.6 (6.6)	6.3	±1.7 (5.4)	6.3	±2.0 (5.7)	0.74	0.63	0.85
Diastolic function											
E wave, cm/s	T1	70.2	±7.4 (1.5)		71.0	±10.5 (2.2)	71.3	±11.8 (0.9)			
	T2	71.7	±7.4^**^ (1.2)		69.5	±${\mathrm{10.1}}^{*}$ (0.8)	69.0	±11.0^**^ (1.3)	0.01	<0.01	0.11
A wave, cm/s	T1	76.5	±12.0 (1.0)		77.3	±13.5 (1.7)	77.1	±25.8 (0.9)			
	T2	75.6	±12.8 (1.6)		77.8	±13.2 (1.1)	76.0	±19.9 (1.5)	0.65	0.59	0.72
E/A	T1	0.9	±0.2 (1.3)		0.8	±0.2 (1.9)	0.9	±0.3 (0.9)			
	T2	1.0	±0.2 (1.5)		0.9	±${\mathrm{0.2}}^{*}$ (1.0)	1.0	±0.2 (1.4)	0.83	0.76	0.81
Septal e’, cm/s	T1	10.5	±2.0 (2.2)		9.1	±2.1 (1.8)	9.0	±2.1 (3.0)			
	T2	10.6	±2.2 (3.1)		9.3	±${\mathrm{2.3}}^{*}$ (1.3)	9.5	±2.1^**^ (1.7)	0.04	0.03	0.19
E/e’	T1	6.6	±2.0 (1.7)		7.7	±3.0 (2.0)	7.8	±2.8 (2.1)			
	T2	6.4	±1.9 (1.9)		7.6	±2.3 (1.1)	7.4	±2.6 (1.4)	0.17	0.28	0.34
DT, ms	T1	213.9	±43.2 (0.9)		207.6	±51.5 (1.1)	210.4	±47.7 (1.2)			
	T2	213.8	±46.8 (0.7)		211.8	±48.7^**^ (0.9)	216.5	±49.6^**^ (1.3)	<0.01	<0.01	0.57
IVRT, ms	T1	80.6	±15.6 (2.3)		77.9	±13.6 (3.2)	83.4	±11.6 (1.6)			
	T2	79.6	±14.8 (1.2)		81.5	±15.5^**^ (3.2)	88.8	±13.6^**^ (2.9)	0.35	0.47	0.51

Data are expressed as mean ± standard deviation and mean error (%). Abbreviations: A wave, diastolic mitral inflow velocity during late atrial contraction; AC, attention control group; CO, cardiac output; DT, deceleration time; E wave, early diastolic mitral inflow velocity; e’, septal mitral annulus early diastolic velocity; GLS, global longitudinal strain; HIIT, high-intensity interval training; ISWT, interventricular septal wall thickness; IVRT, isovolumetric relaxation time; LV, left ventricular; LVEDD, left ventricular end-diastolic diameter; LVEDV, left ventricular end-diastolic volume; LVEF, left ventricular ejection fraction; LVESD, left ventricular end-systolic diameter; LVESV, left ventricular end-systolic volume; LVMI, left ventricular mass index; n, number of patients; PWT, posterior wall thickness; S’, mitral annulus peak velocity in systole; SVI, systolic volume index; T1, baseline measurement; T2, measurement at the end of the intervention (16 weeks). Statistics: Paired test with a two-tailed *p* value was performed to compare the pre-post means of each study group: ^*^:* p *< 0.05 and ^**^:* p *< 0.01 indicate significant differences. Lineal regression was used to compare the delta pre-post main effect (*p*). Time: *p* value indicates the significance of the main effect within the subjects of the same group; Group: *p* value indicates the significance of the main effect between individuals in different groups, and Interaction Group*Time: shows the significance determined by the interaction of the two main effects found. Time: *p* value indicates the main effect of within-group differences. Group:* p* value indicates the main effect of exercise training (AC *vs* HIIT). *p <* 0.05 was considered significant.

Although both HIIT groups showed a mean increase in ventricular mass (low-volume HIIT: 2.2%, high-volume HIIT: 5.7%, *p <* 0.01) and in left ventricle ejection fraction (low-volume HIIT: 3.9%, high-volume HIIT: 4.3%, *p *< 0.05), no significance was found in the analysis of the effect of interventions (*p* = ns). Left ventricular global longitudinal strain (GLS) evidenced a significant reduction in the two HIIT groups at the end of the intervention. The low-volume HIIT group reduced the GLS by –3.2% (*p =* 0.03) while in the high-volume HIIT group the reduction was –4.7% (*p =* 0.018). Meanwhile, E wave showed a –2.1% (*p =* 0.033) and a –3.3% (*p *< 0.01) reduction after low- and high-volume HIIT, respectively. At the same time, the e’ wave increased by 2.2% (*p =* 0.031) in low-volume HIIT and 5.5% (*p =* 0.025) in high-volume HIIT, while deceleration time (DT) increased by 2.1% (*p *< 0.01) in low-volume HIIT *vs.* 2.9% (*p <* 0.01) in high-volume HIIT. The E/A ratio and the isovolumetric relaxation time determination showed significant mean improvements (*p <* 0.01) in the two HIIT groups but not in the effect analysis (Table 3).

### 3.3 Cardiac Biomarkers

**Fig. 2.**
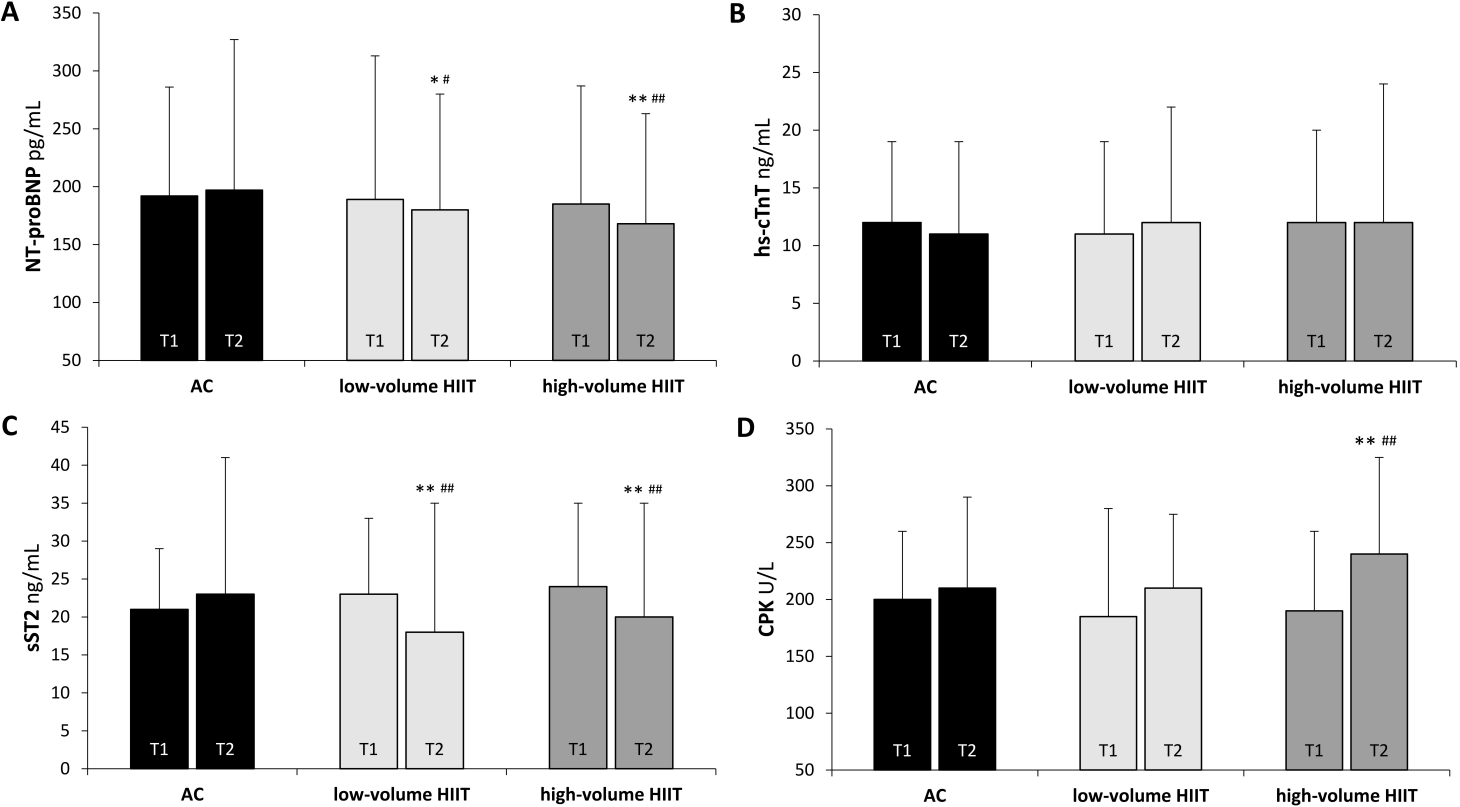
**Changes in cardiac biomarker levels in patients with myocardial infarction after different volumes of aerobic high-intensity interval training (HIIT): low-volume HIIT and high-volume HIIT compared with a control group (AC)**. (A) N-terminal pro-B-type natriuretic peptide (NT-proBNP). (B) High-sensitivity troponin T (hs-cTnT). (C) Soluble ST2 (sST2), and (D) Creatine phosphokinase (CPK). Data are expressed as mean ± standard deviation (error bars). T1, baseline measurement; T2, measurement at the end of the intervention (16 weeks). Statistics: Paired test with a two-tailed *p* value was performed to compare the pre-post means of each study group: *: *p *< 0.05 compared to T1 in the same group; **: *p *< 0.01 compared to T1 in the same group; ^#^: *p *< 0.05 compared to the mean percentage change pre-post (%Δ) of the AC; ^##^: *p *< 0.01 compared to the mean percentage change pre-post (%Δ) of the AC.

Levels of cardiac biomarkers after the different training interventions are shown in Fig. 2. At baseline, there were no significant differences among groups. After 16 weeks of study, the AC did not show significant changes in any of the biomarkers analyzed. However, in the low-volume HIIT, NT-proBNP showed a significant decrease at T2 (–4.8%, *p =* 0.031); while, in the high-volume HIIT, a decrease was also evident (–11.1%, *p <* 0.01). Soluble ST2 levels were significantly reduced in the two HIIT groups compared to the AC group (low-volume HIIT: –21.7%, high-volume HIIT: –16.7%, *p <* 0.01). Furthermore, creatine kinase increased significantly (19.3%, *p <* 0.01) in the high-volume HIIT group after finishing the intervention. Elevated creatine kinase was not related to taking statins or other current medication (*p =* not significant). The hs-TnT showed no changes during all the study times (*p =* 0.411) and correlation analysis between biomarker variations and ultrasound determinations did not show any statistical significance (data not shown).

## 4. Discussion

The present study examined the effect of two different volumes of HIIT compared to AC on echocardiographic and biochemical markers of ventricular remodeling in patients who had recently suffered from MI. The main findings of our research were: (a) globally, HIIT helps by keeping normal ranges of LV remodeling and function compared to an AC group; (b) the main effect on the non-dysfunctional left ventricle focuses on the improvement of diastolic function, and the prevention of deleterious remodeling; (c) HIIT appears to have a preventive volume-dependent effect on remodeling seen at 16 weeks of training although low-volume HIIT could be proposed as a better option than high-volume in post-MI patients.

Within cardiac rehabilitation programs, the beneficial effects of HIIT have been well demonstrated at the peripheral level and less accentuated but significant at the cardiac level compared to moderate-intensity continuous training and control groups [31]. Most of these findings were made in patients with stable CAD [19, 20, 32] and/or heart failure [17, 33]. Focusing on post-MI patients with preserved ventricular function, it has been observed that HIIT can prevent structural reverse remodeling of the ventricle more than its improvement [9, 34]. In the present study, similar findings were observed. The thickness of the ventricular wall remained within normal ranges in the two HIIT groups without evidence of deleterious thinning. This is in line with the growing concept of protection that HIIT has on post-MI ventricular remodeling [33].

Although no significant intervention effect was found on ventricular mass, possibly due to the short intervention time (2 weekly sessions in 16 weeks) [35], significant changes in both LVEDD and LVEDV after the HIIT training period were observed. These results are opposite to those found in another study where post-MI patients with dilated ventricles presented a significant reduction of the diameter and diastolic volume of 12% and 18%, respectively [17]. In contrast, in the present study results are more similar to studies in healthy people (no CAD or comorbidities) or with CAD where initially there was no dilation of the cavities and ventricular function was preserved [20, 36, 37]. These changes in ventricular dimensions could be both a post-MI change as well as an acute response to HIIT. Given the results from the present study, the cardiac adaptation generated by HIIT could attenuate the tendency towards dysfunctional remodeling associated with improvement in biomarkers of myocardial stress (*i.e.*, NT.proBNP and sST2) [38].

The diastolic adaptation, presented by the left ventricle and evidenced by the changes in septal e’, E wave, and DT due to the different HIIT volumes, supports the results of previous studies [32]. This occurs in part because high-intensity exercises place a greater load on the central part of the circulation, inducing large cardiac adaptations [39]. An incremented preload because of plasma volume expansion most probably explains the increase in load-dependent E together with the transient increase in the diastolic size of the left ventricle [40]. Thus, the increase of the ventricular chamber seen in the HIIT groups is explained by the increase of the venous return to the heart, in accordance with the Frank-Starling law, and consequently increased stretching of the LV muscle fibers and LVEDV. As a result, in the HIIT groups, there would be an improvement in LV function mainly due to greater angiogenesis and an increase in the early LV filling time (E wave) [41]. This finding tends to occur similarly in athletes immediately after training [42, 43]. Strikingly, the changes in the two HIIT groups generated significant pre-post differences in E and e’, but not in the E/e’ index. Analyzing the changes of E/e’, reductions of between 1–5% are evidenced, but they are not statistically significant (*p =* 0.053). Considering that it may be the most reliable parameter of diastolic function, it can be assumed that perhaps the method and the small sample of the study did not reach sufficient statistical power. Overall, this highlights the effect of the different HIIT volumes on diastolic function, something that is consistent with previous studies carried out in patients with CAD with and without ventricular dysfunction [31, 32].

On the other hand, the determinations of the systolic function also respond to HIIT although in a less marked way than the diastolic function. Ejection fraction improvement induced by HIIT has already been demonstrated in previous studies and could be related to an attenuation of negative remodeling and an increase in ventricular compliance [17, 37]. To this effect, the left ventricle longitudinal strain featured in GLS, as an important predictor of ventricular remodeling, has been shown to improve with HIIT [20, 44]. On the contrary, and possibly due to the reduced sample size used, previous studies have not observed the same effect [45].

Low-volume appears to be more time-effective than high-volume HIIT, despite many of the beneficial effects being volume-dependent [12], as we have seen in the present study. Previous publications endorse low-volume HIIT, evidencing an increase in mitochondrial capacity in the peripheral striated muscle [46]. Many of the observed ventricular remodeling changes can be considered as good enough already at low-volume HIIT. Adding to this, the potential risk of muscle damage that can be produced by high volumes of HIIT (*i.e.*, creatine kinase elevation) should be noted [47]. Jointly, the proposal that initially a low-volume HIIT is better than a high-volume is being consolidated. Despite the pre-post changes in the two HIIT groups being mostly within normal ranges, what is relevant is that the AC either did not improve or worsened the ventricular parameters analyzed. Clinically, this is relevant to emphasize the importance of including at least a low-volume HIIT intervention in post-MI cardiac rehabilitation programs.

It should be mentioned that the practice of HIIT could present a high joint impact and difficulties to reach the proposed intensity target, which could interfere with adherence and the acquisition of all the known physiological adaptations [48]. For this reason, it is worth noting that the present study is the first to use a mixed bicycle and treadmill model in an attempt to reduce the high demand by HIIT on the joint system and improve its progressive tolerance. Future research should be considered based on the results of this study. The relationship between ventricular changes and delta ${\mathrm{VO}}_{\text{2peak}}$, the effects of detraining, and the impact of different volumes of HIIT on mortality could provide insight into the clinical impact in post-MI patients.

## 5. Limitations 

Our findings must be interpreted in the context of several limitations. First, the sample size in the present study was small with a predominance of men from one single hospital. As such, the possibility of a type 2 error is high. Secondly, specific information on LV dysfunctional regions was not collected, losing the possibility of assessing the impact of HIIT. Thirdly, the systolic and diastolic functions were measured at rest without being able to obtain information on the reserve contractility of the LV during exercise. Fourth, ventricular structure and function were measured only by echocardiography and not by other more sensitive techniques (*i.e*., cardiac magnetic resonance). Finally, the supervised intervention studies with exercise have limited external validity to extend the results to the unsupervised population.

## 6. Conclusions

In patients with MI, HIIT shows a beneficial effect by preventing thinning of the ventricular walls and improving ventricular function, mainly diastolic. These adaptations appear to be dependent on the volume of training performed. Despite this, low-volume HIIT (*i.e*., 20 min of total volume with less than 10 min at a high intensity) is proposed as a clinically time-efficient and safer strategy to attenuate dysfunctional ventricular remodeling in post-MI patients.

## Data Availability

The datasets used and/or analyzed during the current study are available from the corresponding author on reasonable request.
